# Adaptive regression model for Parkinson’s disease diagnosis from speech signals using Box-Cox-based clustering and extremely randomization

**DOI:** 10.1038/s41598-026-49065-2

**Published:** 2026-05-02

**Authors:** Mahmoud Essam, Mazen Balat, Ahmed B. Zaky, Mervat Samy, Ahmed M. Anter

**Affiliations:** 1 Computers and Data Science, Alexandria University, Alexandria, 21526 Egypt; 2 Egypt-Japan University of Science and Technology (E-JUST), New Borg El-Arab, Alexandria 21934 Egypt; 3 Department of Electrical Engineering, Faculty of Engineering (Shoubra), Benha University, Benha, 13511 Egypt; 4 Clinical Pharmacology, Faculty of Medicine, Mansoura University, Mansoura, 35516 Egypt; 5 Faculty of Computers and Artificial Intelligence, Beni-Suef University, Benisuef, 62511 Egypt

**Keywords:** Parkinson’s disease diagnosis, Machine learning, Clustering-based feature selection, Extra trees regressor, AdaBoost regressor, Autoencoders, Computational biology and bioinformatics, Diseases, Mathematics and computing, Neurology, Neuroscience

## Abstract

Parkinson’s Disease (PD) is a progressive neurodegenerative disorder that causes motor and cognitive impairments, affecting approximately 1% of individuals over 60 years of age. Speech impairments are among the earliest and most accessible biomarkers, making voice-based assessment a promising avenue for remote PD monitoring. However, existing speech-based PD prediction methods suffer from feature redundancy that degrades model performance, non-Gaussian data distributions that violate model assumptions, and limited systematic feature grouping strategies. This study introduces an adaptive approach to improve PD diagnostic precision by predicting the Motor Unified PD Rating Scale (UPDRS) and Total-UPDRS scores from biomedical voice measurements. The proposed framework addresses these challenges through three integrated components: (1) Box-Cox transformation to stabilize variance, reduce skewness, and normalize features; (2) a clustering-based feature selection method that groups correlated features via K-Means and selects the most informative representative per cluster using mutual information, thereby eliminating redundancy without losing discriminative power; and (3) an Extra Trees Regressor (ETR) whose extreme randomization in node splitting provides computational efficiency and reduced variance. To ensure rigorous evaluation, a subject-independent data splitting strategy is adopted to prevent data leakage, and *k*-fold cross-validation is employed to assess model stability. The proposed method is compared against multiple feature selection techniques—mutual information, recursive feature elimination, Lasso regression, and autoencoders—paired with nine regression models including Ridge, Lasso, Linear, Decision Tree, k-Nearest Neighbors, Random Forest, Gradient Boosting, AdaBoost, and Extra Trees Regressors. The clustering-based feature selection combined with ETR yielded the best performance, achieving $R^2$ scores of 0.999 for Motor-UPDRS and 0.997 for Total-UPDRS on the test set. These results are further supported by cross-validation analysis and feature importance evaluation, demonstrating the effectiveness and robustness of the proposed framework for speech-based PD telemonitoring.

## Introduction

Parkinson’s disease (PD) is a progressive neurodegenerative disorder primarily marked by the loss of dopaminergic neurons in the substantia nigra, leading to motor symptoms such as tremors, rigidity, bradykinesia, and postural instability^1^. As the second most common neurodegenerative disorder after Alzheimer’s disease, PD affects millions worldwide with a prevalence of approximately 1% in individuals over 60 years of age, and cases are expected to double by 2030 due to an aging population^2^. Early diagnosis is crucial for enabling neuroprotective therapies and improving patient outcomes^3^, yet current diagnostic methods remain challenging due to the lack of definitive biomarkers and reliance on clinical symptoms that typically manifest after significant neuronal damage^4,5^.

Among the various diagnostic modalities, speech-based analysis has emerged as a particularly promising non-invasive approach for PD assessment. Voice impairments—including changes in jitter, shimmer, and harmonic-to-noise ratio—are among the earliest symptoms of PD and can be captured remotely through telemonitoring devices^6^. Several machine learning approaches have been proposed for speech-based PD diagnosis, including deep learning models^7^, support vector machines^8^, and ensemble methods^9^. However, existing approaches face several key limitations: (1) deep learning models require large, high-quality datasets and are computationally intensive, making them difficult to deploy in clinical settings^10,11^; (2) most methods do not systematically address feature redundancy inherent in speech measurements, where multiple jitter and shimmer variants are highly correlated; and (3) many studies lack rigorous validation strategies, particularly regarding subject-independent evaluation to prevent data leakage^12^.

Feature selection is critical for addressing the redundancy problem. Existing feature selection methods—including filter methods (e.g., mutual information), wrapper methods (e.g., recursive feature elimination), and embedded methods (e.g., Lasso regression)—each have limitations^13^. Filter methods are computationally efficient but model-independent, wrapper methods are computationally expensive, and embedded methods are tied to specific learning algorithms. Recently, clustering-based feature selection approaches have gained attention for their ability to group correlated features and select diverse representatives, potentially capturing the data structure more effectively^14,15^.

This paper addresses the aforementioned challenges by proposing an adaptive machine learning framework that integrates three key components specifically designed for speech-based PD diagnosis. First, Box-Cox transformation is applied to normalize the highly skewed voice measurements, ensuring they meet the distributional assumptions of downstream models. Second, a clustering-based feature selection method is proposed that automatically groups correlated speech features using K-Means clustering and selects the most informative feature per cluster via mutual information scoring—this systematically eliminates redundancy while preserving discriminative information. Third, the Extra Trees Regressor (ETR) is employed as the predictive model due to its extreme randomization in split selection, which provides reduced variance and computational efficiency compared to standard Random Forests^16^. The rationale behind this specific combination is that: (a) Box-Cox transformation addresses the non-Gaussian distributions typical of speech biomarkers; (b) clustering-based selection handles the high inter-feature correlation among jitter/shimmer variants; and (c) ETR’s inherent randomization complements the reduced feature space by mitigating overfitting risks. Furthermore, recent advances in machine learning for complex system modelling^17,18^ have demonstrated the effectiveness of ensemble and adaptive techniques in handling high-dimensional biomedical data, motivating the adoption of such strategies in our framework.

The rest of this paper is structured as follows: “Related works” reviews related works in the field, focusing on existing diagnostic methods and their limitations. “Materials and methods involved” details the materials and methodology used in our study, including datasets, machine learning models, and feature selection techniques. “Experimental results and discussion” presents the results of our experiments, including the performance of our model in comparison to traditional diagnostic methods. Finally, “Conclusion and future work” concludes the paper with a discussion of the implications of our findings and potential future research directions.

## Related works

Numerous studies have explored various methodologies for detecting and predicting the severity of Parkinson’s Disease (PD). In this section, we categorize existing research into three main groups: (1) speech and voice-based methods, (2) medical imaging-based methods, and (3) hybrid and telemonitoring-based approaches. For each category, we summarize key findings and analyze their limitations.

### Speech and voice-based methods

Speech analysis has become a prominent non-invasive approach for PD detection due to the strong correlation between voice characteristics and motor impairment.

Changqin et al. utilized speech samples from individuals with PD and healthy controls (HC), applying a Bidirectional Long Short-Term Memory (LSTM) model to dynamic articulation transition features. Their method achieved 96.8% and 94.3% accuracy under different evaluation methods^7^. However, the approach relies on deep learning, which requires substantial computational resources and large datasets. Sanjana et al. leveraged the mPower study dataset, employing machine learning models to detect PD through voice samples, achieving 99.0% accuracy^19^. While impressive, the study did not address potential subject overlap between training and test sets. Arora et al. analyzed sustained vowel phonations from the Parkinson’s Voice Initiative, achieving balanced accuracy of 67.34%^20^, highlighting the challenges of generalization in voice-based PD detection. Liaqat et al. integrated feature selection with deep neural networks using vowel phonation datasets, enhancing PD detection accuracy by 6.5% through an ensemble approach^8^. Annalisa et al. examined handwriting data from PD patients and healthy controls, achieving over 90% accuracy, demonstrating the potential of motor signal analysis for PD telemonitoring^21^.

### Medical imaging-based methods

Medical imaging provides direct visualization of structural and functional brain changes associated with PD.

Saurav et al. developed deep learning models enhanced with the Grey Wolf Optimization (GWO) algorithm for MRI and SPECT DaTscan datasets, achieving near-perfect accuracy with AUC-ROC scores approaching 100^22^. However, imaging-based methods are expensive, require specialized equipment, and are not suitable for continuous remote monitoring. Çağatay et al. used 3D T1-weighted MRI data with 2D and 3D CNNs, achieving an accuracy of 0.9620 and $R^2$ of 0.8372 for severity prediction^23^. While effective, the computational complexity and preprocessing requirements limit scalability for real-time applications.

### Hybrid and telemonitoring-based approaches

Telemonitoring methods combine feature engineering with machine learning to enable continuous remote PD assessment.

María et al. implemented MLPs and autoencoders on the Parkinson’s Telemonitoring Dataset, achieving a 99.15% classification success rate^24^. Aryan et al. employed clustering algorithms with a multilayer neural network on the Oxford Telemonitoring Dataset, achieving 94.4% accuracy^25^. Hyunsoo et al. explored transfer learning for PD telemonitoring, with their Positive Transfer Learning method achieving superior accuracy compared to traditional approaches^26^. Pechprasarn et al. applied 26 machine learning models with PCA on the UCI Parkinson’s Telemonitoring dataset, with the Fine Tree model achieving $R^2$ of 0.90 for Motor-UPDRS and 0.94 for Total-UPDRS^27^. Mehrbakhsh et al. developed a hybrid SVD-ANFIS method, significantly improving accuracy and computation time^28^. Waleed et al. used clustering and ANFIS models, achieving the best predictive performance with low RMSE and $R^2$ values of 0.9876 and 0.9911 for Total- and Motor-UPDRS respectively^29^. Zaifa et al. introduced a patient-specific transfer method using Shapley values for UPDRS prediction^30^. Anter et al. proposed a robust regression model combining BALO feature selection with DEELM prediction, achieving correlation coefficients of approximately 0.908 and 0.912^9^.

The key findings from various studies on Parkinson’s disease detection and severity prediction using different datasets and methodologies are summarized in Table 1 below.

### Limitations of existing studies

Despite the progress made, several critical limitations persist across existing studies:

1. Many speech-based approaches do not adequately address the high correlation among voice features (e.g., multiple jitter and shimmer variants), leading to redundant feature spaces that can degrade model performance.
2. Most studies employ random data splitting at the recording level rather than patient-level splitting, which can introduce data leakage when multiple recordings from the same patient appear in both training and test sets, artificially inflating performance metrics.
3. Deep learning approaches, while powerful, require large datasets and substantial computational resources, limiting their applicability for real-time telemonitoring.
4. Few studies provide comprehensive feature importance analysis or clinical interpretation of their results.
5. The lack of systematic comparison between different feature selection paradigms (filter, wrapper, embedded, and clustering-based) makes it difficult to determine the most effective approach for PD speech data.

**Table 1:** Summary of Parkinson’s disease detection and severity prediction studies.

Authors	Datasets	Methodology	Results
Changqin Quan et al.^7^	Speech samples from PD and HC	Bidirectional LSTM model on dynamic articulation transition features	96.8% accuracy (10-fold CV), 94.3% accuracy (split dataset)
María Teresa García-Ordás et al.^24^	Parkinson’s telemonitoring dataset	Max–min normalization, MLPs for classification and regression, autoencoders for dimensionality reduction	99.15% success rate in classification, MSE of 0.15 in prediction
Aryan Vats et al.^25^	Parkinson’s disease: Oxford Telemonitoring Dataset	Preprocessing for missing values, normalization, clustering algorithms, multilayer neural network	94.4% accuracy, loss of 0.1146
Hyunsoo Yoon et al.^26^	Telemonitoring for PD using AHTD	Transfer learning (PTL) method, predictive analytics	Significantly better accuracy compared to single learning and one-model-fits-all approaches
Saurav Mallik et al.^22^	T1, T2-weighted MRI and SPECT DaTscan datasets	Preprocessing MRI images, deep learning models enhanced with GWO algorithm	Near or above 99% accuracy, AUC-ROC score of 99.99 for T1, T2-weighted dataset, 100 for SPECT DaTscan dataset
Çağatay Berke Erdaş et al.^23^	T1-weighted MRI	FLIRT image registration, BET non-brain tissue scraping, 2D and 3D CNNs	Accuracy of 0.9620, Recall of 0.9536, R of 0.9150, $\textrm{R}^{2}$ of 0.8372
Pechprasarn et al.^27^	UCI Parkinson’s telemonitoring dataset	Data curation, feature selection, 26 machine learning models, PCA	Fine tree model: RMSE of 2.55, R-squared of 0.90 for motor-UPDRS, RMSE of 2.65, R-squared of 0.94 for total-UPDRS
Mehrbakhsh Nilashi et al.^28^	Parkinson’s telemonitoring dataset	SVD for dimensionality reduction, clustering techniques, ensembles of ANFIS	Significant improvement in accuracy and computation time
Waleed Abdu Zogaan et al.^29^	Parkinson’s telemonitoring dataset	Preprocessing data, clustering using OLVQ1, ANFIS models	Best predictive performance with Gaussian MF: RMSE values (UPDRS (total)=0.5732; UPDRS (motor)=0.5645), R-squared values (UPDRS (total)=0.9876; UPDRS (motor)=0.9911)
Annalisa Mancini et al.^21^	Dataset of 16 PD patients and 42 healthy controls	Extracting features from graphic tablet signals, statistical selection, machine learning models	High accuracy exceeding 90% for some models
Zaifa Xue et al.^30^	Parkinson’s telemonitoring dataset	Preprocessing voice data, subject transfer mechanism, Shapley value, random forest model	Lower mean absolute error, root mean square error, and volatility in predicting motor-UPDRS and total-UPDRS scores
Sanjana Singh et al.^19^	mPower study dataset	Preprocessing 10-second audio clips, extracting audio features, machine learning models	Accuracy of 99.0% in under a second
Arora et al.^20^	Parkinson’s Voice Initiative dataset	Preprocessing phonations, extracting dysphonia measures, feature selection and transformation, machine learning models	Balanced accuracy of 67.34% with 67.43% sensitivity and 67.25% specificity
Ahmed M. Anter et al.^9^	Tele-monitoring PD dataset	Preprocessing dataset, BALO for feature selection, DEELM for continuous prediction	High prediction accuracies for motor and Total-UPDRS scores, correlation coefficients of approximately 0.908 and 0.912
Liaqat Ali et al.^8^	Two vowel phonations datasets	Feature extraction using Praat software and MFCC, feature selection using F-score, DNN and conventional machine learning models	Feature selection integration with DNN outperformed conventional models, EOFSC ensemble model enhanced PD detection accuracy by 6.5%

This paper addresses the identified limitations by:

1. Applying Box-Cox transformation to normalize skewed speech features.
2. Proposing a clustering-based feature selection method that systematically handles feature redundancy.
3. Employing subject-independent data splitting to prevent data leakage.
4. Conducting comprehensive comparisons across multiple feature selection paradigms and regression models.

The integration of these components results in a robust and accurate framework for predicting PD severity from speech signals.

## Materials and methods involved

This section details the materials and methodologies used in this study to predict the severity of Parkinson’s disease symptoms by analyzing biomedical voice measurements. We will walk through the dataset used, the steps taken to preprocess the data, the techniques for feature extraction, and the regression models applied. In addition, the evaluation metrics and cross-validation methods that are crucial in assessing the effectiveness of this models are presented.

### Dataset acquisition and description

#### Dataset acquisition

The phonations were recorded in an IAC sound-treated booth using a head-mounted microphone (AKG C420) positioned at 8 cm from the lips. The microphone was calibrated using a class 1 sound level meter (B&K 2238) placed 30 cm from the speaker. The voice signals were recorded directly to the computer using CSL 4300B hardware and sampled at 44.1 kHz with 16-bit resolution^6,9^.

#### Dataset description

For this study, the Oxford Parkinson’s Disease Dataset has been utilized, a rich collection of biomedical voice measurements carefully gathered to help predict the severity of Parkinson’s disease symptoms. This dataset is the result of a collaboration between Athanasios Tsanas and Max Little from the University of Oxford, along with 10 medical centers across the United States, and Intel Corporation^6^. The telemonitoring device used, developed by Intel, allowed for the non-invasive and remote recording of speech signals, making it possible to continuously monitor the progression of symptoms in Parkinson’s patients.

The dataset consists of 5875 instances from 28 males and 14 women, featuring 26 attributes, all derived from voice recordings of 42 individuals diagnosed with early-stage Parkinson’s disease. Each patient contributed around 200 recordings over a 6-month period, which were captured automatically in their homes. This approach ensured that the data reflected real-world conditions, providing a more accurate picture of the disease’s progression. The total-UPDRS range from 0–176 (0 means healthy and 176 means total impairment) and the motor-UPDRS from 0–108 (0 means healthy and 108 means serious motor impairment). The primary goal of this dataset is to support regression tasks, particularly in predicting the motor Unified Parkinson’s Disease Rating Scale (UPDRS) and Total-UPDRS scores based on the biomedical voice measurements.

The dataset includes a variety of voice measures, such as:

- Multiple metrics for variation in fundamental frequency (e.g., Jitter(%), Jitter(Abs), Jitter:RAP, Jitter:PPQ5, Jitter:DDP)
- Several measures for variation in amplitude (e.g., Shimmer, Shimmer(dB), Shimmer:APQ3, Shimmer:APQ5, Shimmer:APQ11, Shimmer:DDA)
- Two measures that quantify the ratio of noise to tonal components in the voice (NHR, HNR)
- A nonlinear dynamical complexity measure (RPDE)
- A signal fractal scaling exponent (DFA)
- A nonlinear measure of fundamental frequency variation (PPE)

The dataset also includes a status of each subject that indicates the health condition, where “0” represents a healthy individual and “1” represents someone with Parkinson’s disease. In the proposed approach, two critical features are of particular importance: motor-UPDRS and total-UPDRS. The motor-UPDRS column records the motor examination score, reflecting the severity of motor symptoms, while the total-UPDRS column provides a comprehensive measure of the overall severity of PD symptoms.

This study aims to develop an adaptive machine learning model based on Box-Cox transformation and Clustering-based speech feature selection to predict the Parkinson’s disease through motor and total-UPDRS scores using the biomedical voice measures. By employing sophisticated feature extraction techniques and advanced machine learning models, we strive to create robust model for monitoring and assessing PD, with the ultimate goal of improving patient care and disease management.

**Fig. 1:**
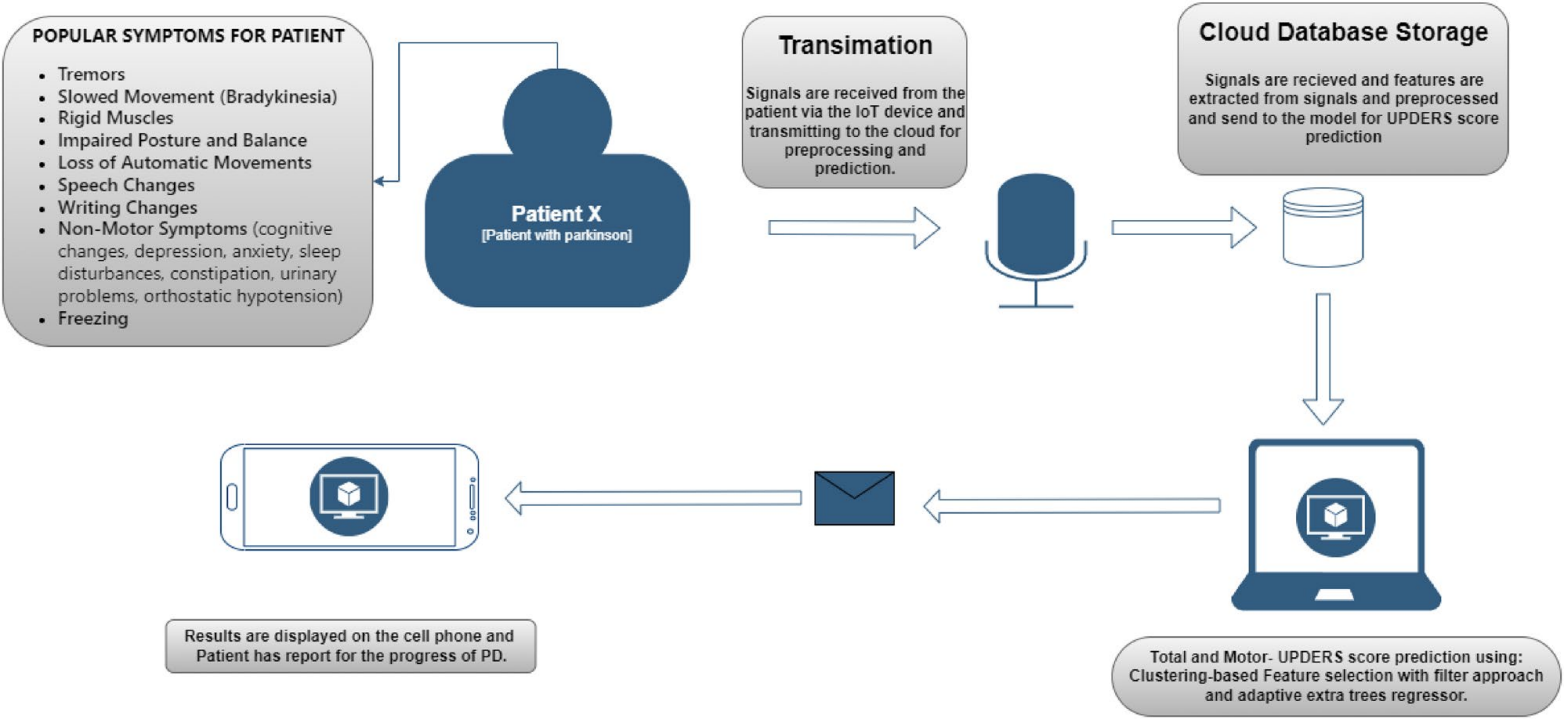
Workflow diagram depicting the integration of IoT and machine learning methods for monitoring and predicting Parkinson’s disease symptoms, with results displayed via a mobile app.

Furthermore, a comprehensive workflow diagram is presented that demonstrates how IoT-based cloud framework and machine learning can be integrated to monitor and predict symptoms in Parkinson’s patients. This diagram illustrates the process, from data collection via IoT devices to symptom prediction using adaptive machine learning model-based cloud computation framework, and the display of results through a mobile app. This workflow provides a clear and efficient pathway for continuous monitoring and management of Parkinson’s disease symptoms as shown in Fig. 1.

### Data preprocessing

Data preprocessing is a crucial step in developing machine learning models, as it ensures that the data used for training is of high quality and reliability^31^. In this study, we undertook several preprocessing steps to prepare the dataset for diagnosing Parkinson’s disease.

**Fig. 2:**
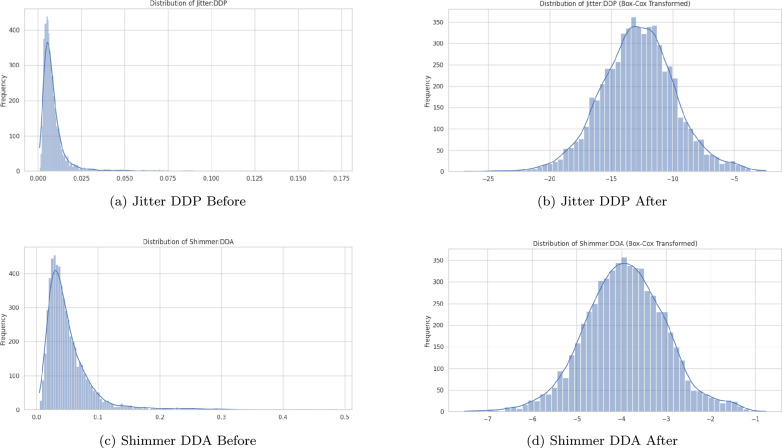
Distribution of columns before and after Box-Cox transformation.

The first step in the preprocessing pipeline is to check for any missing values in the dataset. Missing data can significantly affect the performance of machine learning models, introducing biases or reducing the accuracy of predictions. Then, Normalization is another essential preprocessing step that helps bring all the features onto a common scale, ensuring that each feature contributes equally to the model’s performance. For this study, the Box-Cox transformation is applied. The Box-Cox transformation is a powerful technique that stabilizes variance and helps in making the data more normally distributed^32^. The method uses a parameter called lambda, which decides how the data is transformed. The best value for lambda is usually found automatically. By applying this transformation, we aimed to enhance the performance of the machine learning model by reducing the impact of outliers and skewness in the data. Figure 2 shows the distribution of two features before and after applying the Box-Cox transformation.

Another important step in our preprocessing process is shuffling the data. Shuffling ensures that the training, validation, and test sets are representative of the entire dataset. Randomly shuffling the data helps prevent biases that might arise from the order in which the data is collected. This step is particularly crucial for time-series data or data collected in a specific sequence. By shuffling the data, a more robust and generalizable model is created which capable of performing well on unseen data.

The final step in our preprocessing pipeline is to split the dataset into training, validation, and test sets. Critically, since the dataset contains multiple recordings per patient (approximately 200 recordings per subject), a subject-independent (patient-level) splitting strategy is adopted to prevent data leakage. Specifically, the 42 subjects are first divided into non-overlapping groups for training, validation, and testing, ensuring that all recordings from a given patient appear exclusively in one partition. This prevents the model from learning patient-specific patterns during training that would be encountered during testing, which would artificially inflate performance metrics. This division is essential for accurately evaluating the performance of the machine learning models. The training set is used to train the models, the validation set to fine-tune hyperparameters and prevent overfitting, and the test set to evaluate the final model performance. A standard split ratio of approximately 64% for training, 16% for validation, and 20% for testing is adopted at the subject level. Additionally, all preprocessing steps—including Box-Cox transformation and feature selection—are performed exclusively on the training data to prevent any indirect data leakage. The validation set is used to fine-tune hyperparameters, and the test set is reserved for final model evaluation. This rigorous splitting strategy ensures that the reported performance metrics reflect genuine generalization capability.

### Feature extraction from voice signals

Features extraction from voice signals are a vital step in converting raw audio data into numerical features that can be fed into machine learning models. This process involves analyzing the voice signals to extract features that capture the essential characteristics of speech, especially those influenced by PD.

Acoustic features quantify the variations in frequency and amplitude within the voice signal^33^. Jitter, which measures the cycle-to-cycle variation in the pitch period (fundamental frequency $F_0$), is calculated as:

1 $$\begin{aligned} \text {Jitter}(\%) = \frac{1}{N-1} \sum _{i=1}^{N-1} \left| \frac{T_i - T_{i+1}}{T_i} \right| \times 100, \end{aligned}$$

where $T_i$ and $T_{i+1}$ are consecutive pitch periods.

Shimmer measures the variability in the amplitude of successive speech cycles and is given by:

2 $$\begin{aligned} \text {Shimmer}(\%) = \frac{1}{N-1} \sum _{i=1}^{N-1} \left| \frac{A_i - A_{i+1}}{A_i} \right| \times 100, \end{aligned}$$

where $A_i$ and $A_{i+1}$ are the amplitudes of consecutive speech cycles.

The Harmonics-to-Noise Ratio (HNR) quantifies the ratio of periodic (harmonic) components to noise in the voice signal:

3 $$\begin{aligned} \text {HNR (dB)} = 10 \times \log _{10} \left( \frac{\text {Power of Harmonics}}{\text {Power of Noise}} \right) . \end{aligned}$$

Prosodic features capture the rhythm, stress, and intonation patterns in speech^34^. The fundamental frequency $F_0$ represents the lowest frequency of a periodic waveform, essentially the pitch of the voice. Intensity refers to the loudness of the speech, calculated as:

4 $$\begin{aligned} \text {Intensity (dB)} = 10 \times \log _{10} \left( \frac{\text {Power of Signal}}{\text {Reference Power}} \right) . \end{aligned}$$

Nonlinear dynamical features describe the complexity and irregularities in the voice signal, which are particularly relevant for detecting the effects of PD^35^. Recurrence Period Density Entropy (RPDE) measures the complexity of the signal by analyzing the recurrence of similar states in the signal’s phase space. Detrended Fluctuation Analysis (DFA) assesses long-range correlations in time series data and is defined by the scaling exponent $\alpha$, extracted from the relation:

5 $$\begin{aligned} F(n) \sim n^{\alpha }, \end{aligned}$$

where $F(n)$ is the fluctuation function over a window of size $n$.

Pitch period entropy (PPE)^36^ measures the variability and unpredictability in the pitch periods, given by:

6 $$\begin{aligned} \text {PPE} = -\sum p_i \log (p_i), \end{aligned}$$

where $p_i$ represents the probability of occurrence for each pitch period.

These features are extracted from the original voice signals, converting them into a structured, tabular format suitable for machine learning model. This step is crucial for transforming the raw voice data into meaningful and interpretable features for predicting PD symptoms.

**Fig. 3:**
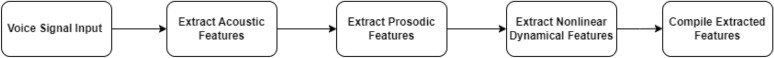
Feature extraction pipeline for voice signals.

The feature extraction process is visually summarized in Fig. 3. This pipeline outlines the various stages, from preprocessing the raw voice signals to extract and organize the features used for machine learning model. The figure demonstrates how acoustic, prosodic, and nonlinear dynamical features are systematically derived and prepared to facilitate the analysis and prediction of PD symptoms.

### Feature selection

Feature selection is a crucial step in the machine learning process as it helps reduce data dimensionality and enhances model performance. In this study, several feature selection techniques are compared and explored to identify the most relevant features for diagnosing Parkinson’s disease. The following are the proposed methods used in this study for feature selection from different feature selection categories (Filter, Wrapper, Embedded, Clustering, and Autoencoder (AE)):

#### Filter method: mutual information

Filter methods focus on selecting features based on their relevance to the target variable, independent of the machine learning algorithm being used. One popular filter method is Mutual Information (MI)^37^, which measures the dependency between two variables. The Mutual Information between two variables $X$ and $Y$ is defined as:

7 $$\begin{aligned} MI(X, Y) = \sum _{x \in X} \sum {y \in Y} P(x, y) \log \left( \frac{P(x, y)}{P(x)P(y)} \right) , \end{aligned}$$

where $P(x, y)$ is the joint probability distribution of $X$ and $Y$, while $P(x)$ and $P(y)$ are their marginal distributions. Features with higher MI values are considered more relevant and are thus selected for model training. MI is particularly effective in capturing non-linear relationships between features and the target variable, making it a powerful tool for feature selection.

#### Wrapper method: recursive feature elimination

Wrapper methods involve evaluating a machine learning model’s performance to select the best subset of features. A well-known wrapper method is Recursive Feature Elimination (RFE)^38^, which iteratively removes the least important features based on the model’s performance. RFE can be used with various algorithms, such as linear regression or support vector machines (SVMs). The importance of a feature $f_i$ is typically determined by the model’s coefficients or weights. The process of RFE can be summarized as follows:

1. Train the model on the initial set of features.
2. Rank the features based on their importance.
3. Remove the least important feature.
4. Repeat the process until the desired number of features is achieved.

The iterative nature of RFE ensures that the selected features are optimized for the specific model, potentially leading to better performance than filter methods. However, RFE can be computationally intensive, especially for large datasets.

#### Embedded method: lasso regression

Embedded methods incorporate feature selection into the model training process itself. Lasso Regression is a popular embedded method that adds a regularization term to the linear regression objective function^39^. The Lasso objective function is given by:

8 $$\begin{aligned} \min _{\beta } \left( \frac{1}{2N} \sum _{i=1}^{N} \left( y_i - \beta _0 - \sum _{j=1}^{p} \beta _j x_{ij} \right) ^2 + \lambda \sum _{j=1}^{p} |\beta _j| \right) , \end{aligned}$$

where $N$ is the number of samples, $p$ is the number of features, $y_i$ is the target variable, $x_{ij}$ is the value of the $j$-th feature for the $i$-th sample, $\beta _0$ is the intercept, $\beta _j$ are the coefficients, and $\lambda$ is the regularization parameter. The L1 regularization term $\lambda \sum _{j=1}^{p} |\beta _j|$ encourages sparsity in the coefficients, effectively performing feature selection by setting some coefficients to zero. Lasso Regression is especially beneficial for high-dimensional data, where many features may be irrelevant or redundant.

#### Autoencoder-based feature selection

Autoencoders are neural network-based models designed to learn a compressed representation of input data. An autoencoder consists of two parts: an encoder network that maps the input data to a lower-dimensional latent space, and a decoder network that reconstructs the input data from the latent representation^40^. The autoencoder’s objective is to minimize the reconstruction error between the input $x$ and the reconstructed output $\hat{x}$. The encoder function $f$ and the decoder function $g$ are defined as follows:

9 $$\begin{aligned} z = f(x) = \sigma (Wx + b), \end{aligned}$$

10 $$\begin{aligned} \hat{x} = g(z) = \sigma '(W'z + b'), \end{aligned}$$

where $x$ is the input data, $z$ is the latent representation, $\hat{x}$ is the reconstructed data, $W$ and $W'$ are the weight matrices, $b$ and $b'$ are the bias vectors, and $\sigma$ and $\sigma '$ are the activation functions. The latent representation $z$ serves as the selected features for model training. Autoencoders are particularly effective for non-linear dimensionality reduction and can capture complex patterns in the data.

#### Clustering-based feature selection

Clustering-based feature selection method group similar features together and select representative features from each cluster. This approach helps reduce redundancy and ensures that the selected features capture the underlying structure of the data^14^. Clustering algorithms such as k-means or hierarchical clustering can be used to group features based on their similarity. The process involves the following steps:

1. Cluster the features based on their similarity.
2. Select a representative feature from each cluster.
3. Use the selected features for model training.

Clustering feature selection is particularly useful for high-dimensional datasets, where many features may be correlated^15^. By selecting representative features from each cluster, the dimensionality of the data is reduced while retaining the most relevant information.

By exploring these feature selection techniques, we aimed to compare the proposed method with different methods to identify the most effective one to enhance the performance of machine learning model, particularly for Parkinson’s disease diagnosis. Each method has its strengths and weaknesses depends on the Parkinson’s dataset characteristics. Evaluating these techniques provides a thorough understanding of their impact on model performance and helps guide the selection of the most appropriate feature selection strategy for Parkinson disease diagnosis.

### Regression models

In this study, several regression models are evaluated as baselines to compare against the proposed clustering-ETR framework. For brevity, we provide concise descriptions of each model; detailed mathematical formulations can be found in the cited references. The hyperparameters of all models are tuned via grid search with *k*-fold cross-validation. The following models are considered:

#### Ridge regressor

The ridge regressor is a linear regression model that includes L2 regularization. This model adds a penalty equal to the sum of the squared coefficients to the loss function, helping to prevent overfitting by shrinking the coefficients^41^. The following equation show the ridge regressor prediction:

11 $$\begin{aligned} \hat{y} = {\bf w}^\top {\bf x} + b \end{aligned}$$

The loss function with the L2 penalty is:

12 $$\begin{aligned} \text {Loss} = \frac{1}{2n} \sum _{i=1}^{n} \left( y_i - \hat{y}_i \right) ^2 + \lambda \Vert {\bf w}\Vert _2^2 \end{aligned}$$

where $\lambda$ is the regularization parameter, $\Vert {\bf w}\Vert _2^2$ is the L2 norm of the weight vector.

#### Lasso regressor

The lasso regressor is another linear regression model, but it uses L1 regularization. This model adds a penalty equal to the sum of the absolute values of the coefficients to the loss function, which can result in a sparse model where some coefficients are set to zero^42,43^. The following equation show the lasso regressor prediction:

13 $$\begin{aligned} \hat{y} = {\bf w}^\top {\bf x} + b \end{aligned}$$

The loss function with the L1 penalty is:

14 $$\begin{aligned} \text {Loss} = \frac{1}{2n} \sum _{i=1}^{n} \left( y_i - \hat{y}_i \right) ^2 + \lambda \Vert {\bf w}\Vert _1 \end{aligned}$$

where $\lambda$ is the regularization parameter, $\Vert {\bf w}\Vert _1$ is the L1 norm of the weight vector.

#### Decision tree regressor (DTR)

The Decision Tree Regressor is a non-parametric, supervised learning method used for regression. It splits the data into subsets based on feature values and fits a simple model within each subset^44,45^. The following equation show the DTR prediction:

15 $$\begin{aligned} \hat{y} = \sum _{m=1}^{M} w_m \cdot I({\bf x} \in R_m) \end{aligned}$$

where $M$ is the number of regions, $w_m$ is the predicted value in region $R_m$, $I({\bf x} \in R_m)$ is an indicator function that is 1 if ${\bf x}$ is in region $R_m$ and 0 otherwise.

#### k-Nearest neighbors regressor (K-NNR)

The k-Nearest Neighbors (k-NN) Regressor is a non-parametric method that predicts the target value for a given input by averaging the target values of the k nearest neighbors in the training set^46^. The following equation show the K-NNR prediction:

16 $$\begin{aligned} \hat{y} = \frac{1}{k} \sum _{i=1}^{k} y_{\text {neighbor}_i} \end{aligned}$$

where $k$ is the number of nearest neighbors, $y_{\text {neighbor}_i}$ is the target value of the $i$-th nearest neighbor.

#### Random forest regressor (RFR)

The Random Forest Regressor is an ensemble learning method that builds multiple decision trees during training and outputs the mean prediction of the individual trees^47^. This model introduces randomness by selecting a random subset of features for each tree. The following equation show the RFR prediction:

17 $$\begin{aligned} \hat{y} = \frac{1}{T} \sum _{t=1}^{T} \hat{y}^{(t)} \end{aligned}$$

where $T$ is the number of trees in the forest, $\hat{y}^{(t)}$ is the prediction of the $t$-th decision tree.

#### Gradient boosting regressor (GBR)

The Gradient Boosting Regressor is an ensemble technique that builds models in a sequence, with each new model correcting the errors made by the previous ones^48^. The following equation show the GBR prediction:

18 $$\begin{aligned} \hat{y}_m = \hat{y}_{m-1} + \eta \cdot f_m({\bf x}) \end{aligned}$$

where $\hat{y}_m$ is the prediction of the model after $m$ iterations, $\eta$ is the learning rate, $f_m({\bf x})$ is the weak learner (usually a decision tree) at the $m$-th iteration.

#### AdaBoost regressor (ABR)

The AdaBoost Regressor is another ensemble method that combines multiple weak learners to create a strong learner. It iteratively trains new models to focus on the instances that previous models predicted incorrectly^49^. The following equation show the ABR prediction:

19 $$\begin{aligned} \hat{y} = \sum _{m=1}^{M} \alpha _m h_m({\bf x}) \end{aligned}$$

where $M$ is the number of weak learners, $\alpha _m$ is the weight assigned to the $m$-th weak learner, $h_m({\bf x})$ is the prediction of the $m$-th weak learner.

#### Extra trees regressor (ETR)

ETR is an ensemble machine learning model that combines multiple decision trees. ETR is a variant of the Random Forest algorithm that introduces even more randomness into the model-building process. Unlike Random Forest, it uses random thresholds for each feature rather than searching for the best possible threshold^16^. This makes it particularly attractive for large scale applications where computational efficiency is crucial. Each ETR tree uses the complete training dataset rather than a bootstrap sample. Furthermore, ETR algorithm has the advantages as follows: (a) It works well with missing values and outliers, and (b) can find complex relationships in Parkinson’s data automatically. (c) the node splitting process is very simple and more effective than other ensemble learning methods. (d) ETR algorithm reduces the risk of overfitting compared to standard decision trees due to its high randomization in split selection, which decreases variance at the cost of slightly increased bias. However, it should be noted that ensemble randomness alone does not eliminate overfitting, particularly in datasets with repeated subject measurements; therefore, proper validation strategies such as subject-independent splitting are essential. When other methods get stuck trying to be perfect, ETR method often finds better answers by being more chaotic. The following equation show the ETR prediction:

20 $$\begin{aligned} \hat{y} = \frac{1}{T} \sum _{t=1}^{T} \hat{y}^{(t)} \end{aligned}$$

where $T$ is the number of trees in the ensemble, $\hat{y}^{(t)}$ is the prediction of the $t$-th decision tree.

The model’s strength comes from its extreme randomization and the few key hyperparameters needed for configuration. This additional randomization makes extra trees generally faster to train while maintaining comparable performance to other machine learning methods.

Evaluating these models provided insights into their performance on predicting motor and Total-UPDRS scores, allowing us to compare their strengths and weaknesses for monitoring Parkinson’s disease progression.

### Model evaluation

Evaluating the performance of the propose models is a critical step in the machine learning pipeline, ensuring their reliability and ability to generalize to new data^50^. In this study, a variety of evaluation metrics are used to rigorously assess model accuracy, including Mean Absolute Error (MAE), Mean Squared Error (MSE), Root Mean Squared Error (RMSE), and the coefficient of determination ($\textrm{R}^{2}$). To further validate the models robustness, cross-validation is employed, which helped gauge consistency across different data partitions.

During the training, validation, and testing phases, the following metrics are used to evaluate model performance:

- Mean absolute error (MAE)^51^: MAE provides a straightforward measure of the average magnitude of errors in the predictions, offering a clear interpretation without considering the direction of errors. It can be calculated from the following formula.
  21 $$\begin{aligned} \text {MAE} = \frac{1}{N} \sum _{i=1}^{N} |y_i - \hat{y}_i| \end{aligned}$$
  where $N$ denotes the number of samples, $y_i$ represents the actual observed values, and $\hat{y}_i$ corresponds to the model’s predicted values.
- Mean squared error (MSE)^52^: MSE quantifies the average squared difference between the actual and predicted values, placing greater emphasis on larger errors. This metric is particularly useful for identifying models that might produce occasional but significant prediction errors. It can be calculated from the following formula.
  22 $$\begin{aligned} \text {MSE} = \frac{1}{N} \sum _{i=1}^{N} (y_i - \hat{y}_i)^2 \end{aligned}$$
- Root mean squared error (RMSE)^51^: RMSE, derived from MSE, provides an error metric in the same units as the target variable, making it easier to interpret in the context of the data. It effectively captures the standard deviation of prediction errors, offering insights into the typical magnitude of model errors. It can be calculated from the following formula.
  23 $$\begin{aligned} \text {RMSE} = \sqrt{\frac{1}{N} \sum _{i=1}^{N} (y_i - \hat{y}_i)^2} \end{aligned}$$
- Coefficient of determination ($\textrm{R}^{2}$)^53^: $\textrm{R}^{2}$ evaluates how well the model explains the variance of the dependent variable relative to its mean. A value close to 1 indicates a high degree of explanatory power, while values nearer to 0 suggest a less effective model. It can be calculated from the following formula.
  24 $$\begin{aligned} R^2 = 1 - \frac{\sum _{i=1}^{N} (y_i - \hat{y}_i)^2}{\sum _{i=1}^{N} (y_i - \bar{y})^2} \end{aligned}$$

These metrics collectively provide a comprehensive understanding of the model’s predictive performance, offering multiple perspectives on accuracy, error magnitude, and explanatory power.

**Fig. 4:**
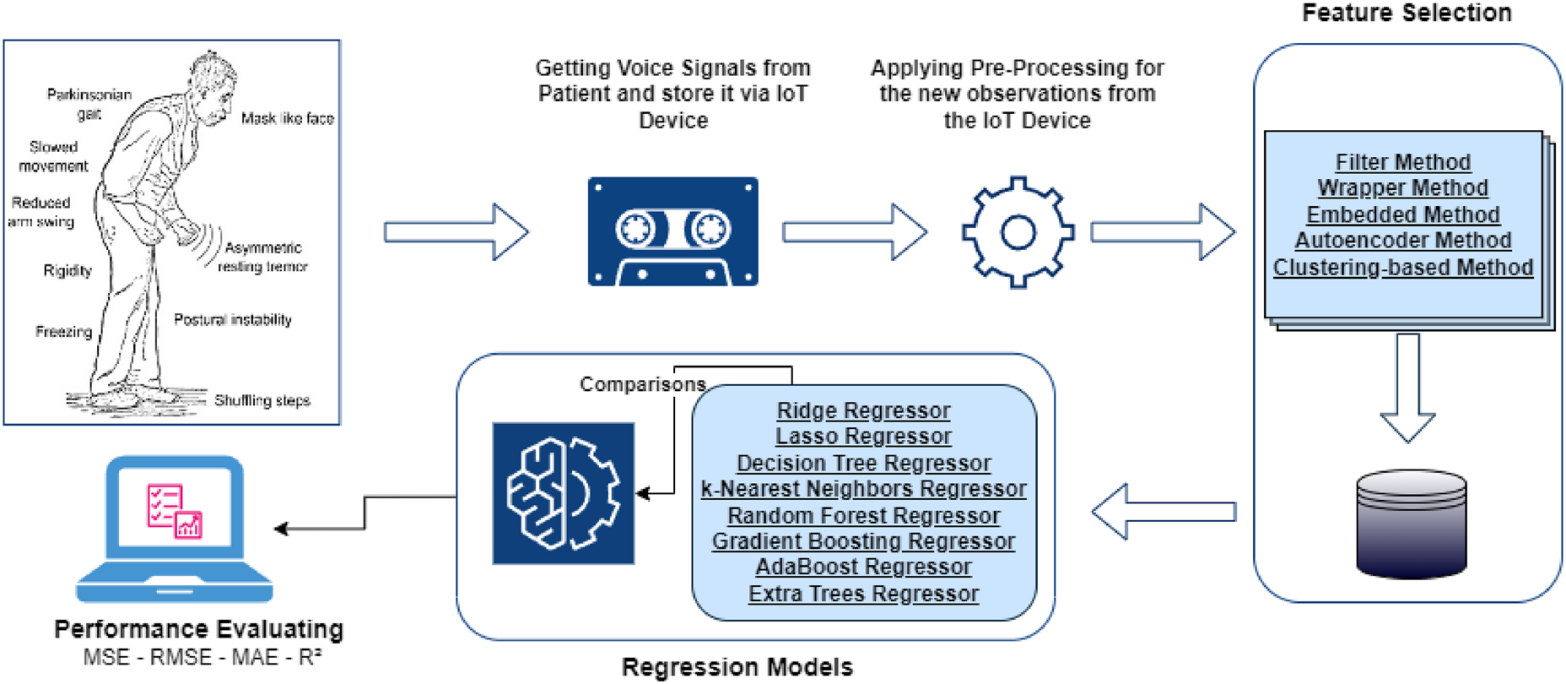
Overall architecture of the proposed method.

In summary, the proposed model is used for predicting the severity of Parkinson’s disease symptoms using biomedical voice measurements involves a comprehensive pipeline, as illustrated in Fig. 4. The pipeline includes data collection and preprocessing, feature extraction, model training and evaluation, and performance assessment using various metrics. By integrating these components, we aim to develop robust and accurate regression model that can effectively predict motor and total-UPDRS scores, thereby enhancing the remote monitoring and assessment of Parkinson’s disease symptoms. This architecture ensures a systematic and rigorous approach to model development and evaluation, ultimately contributing to improved patient care and disease management. Algorithm 1 shows the workflow of the proposed adaptive regression framework for PD diagnosis.

**Algorithm 1:**
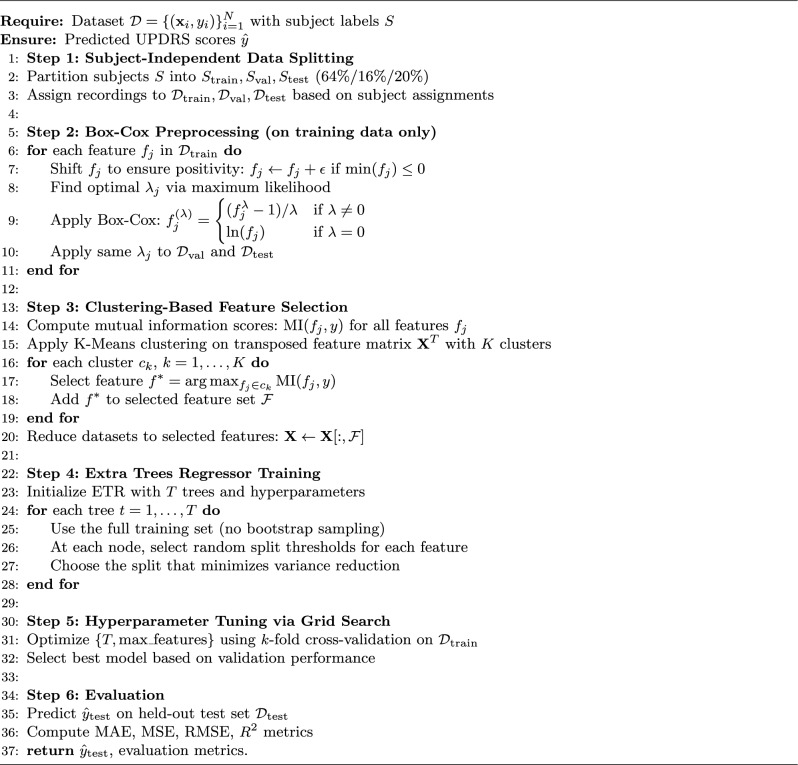
Proposed adaptive regression framework for PD diagnosis.

## Experimental results and discussion

### Experimental design and setup

The experiments were conducted using the Kaggle Service, which provided two NVIDIA Tesla T4 GPUs, each with 15 GB of GPU memory. The session duration was set to a maximum of 12 h, with a disk space allocation of up to 57.6 GiB and 29 GiB of RAM. This setup ensured efficient model training and evaluation.

Table 2 presents the feature selection methods used in the experiments along with their best parameters for achieving the target metrics. The table includes methods such as Filter, Wrapper, Embedded, Clustering, and Autoencoder (AE). Each method is described with its specific parameters for both the Total-UPDRS target and the Motor-UPDRS target. This table provides a comprehensive overview of the feature selection techniques employed and their respective configurations, which were crucial for optimizing the model performance.

**Table 2:** Feature selection methods and their parameters.

Feature selection method	Parameters for total-UPDRS target	Parameters for motor-UPDRS target
Filter	k = 5	k = 10
Wrapper	K = 18	K = 18
Clustering	C = 3	C = 3
Auto Encoder	encoding_dim=10, epochs=100, batch_size=64, target=total_UPDRS	encoding_dim=10, epochs=100, batch_size=32, target=motor_UPDRS

### Mutual information-based filter selection approach

**Fig. 5:**
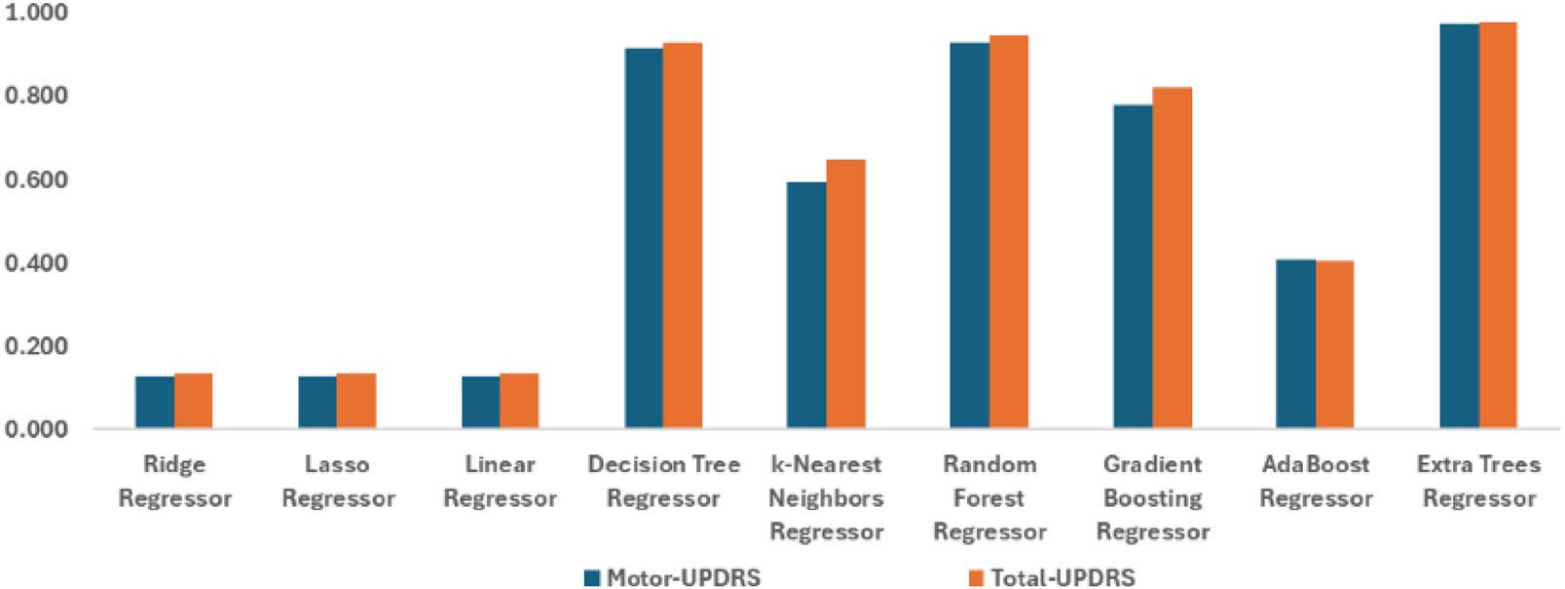
Graphical representation for the performance of filter feature selection approach-based mutual information for motor and total- UPDRS scores under $R^2$ with different machine learning models-based regression.

Figure 5 shows the performance results in predicting Motor- and Total-UPDRS scores. The Extra Trees Regressor stands out, achieving the best performance across all metrics with an $R^2$ value of 0.976 for Motor-UPDRS and 0.978 for Total-UPDRS. The Random Forest Regressor also performed well and closely following the ETR method, though with slightly higher error metrics. It achieved an $R^2$ value of 0.928 for Motor-UPDRS and 0.945 for Total-UPDRS, followed by Decision Tree Regressor with an $R^2$ value of 0.914 for Motor-UPDRS and 0.929 for Total-UPDRS. In contrast, linear models such as Ridge, Lasso, and Linear Regressors demonstrate significantly higher error rates, with $R^2$ values around 0.128, suggesting these models struggle with the non-linear relationships present in the data. For more details see Table 3 that highlight the clear advantage of ensemble methods over conventional methods in predicting Motor- and Total- UPDRS scores. This indicates a strong ability to capture the underlying patterns in the data, leading to highly accurate predictions.

These findings underscore the importance of selecting robust, non-linear model like the Extra Trees Regressor when dealing with complex datasets where capturing intricate interactions is crucial for predictive accuracy.

**Table 3:** Performance comparison of regression models based on filter approach for motor- and total- UPDRS scores.

Regressor	Target	MSE	RMSE	MAE	R^2^
Ridge regressor	Motor-UPDRS	6.621	60.506	7.779	0.128
Lasso regressor		6.634	60.478	7.777	0.128
Linear regressor		6.622	60.52	7.779	0.127
Decision tree regressor		1.61	5.995	2.448	0.914
k-nearest neighbors regressor		2.364	28.181	5.309	0.594
Random forest regressor		1.576	4.985	2.233	0.928
Gradient boosting regressor		3.133	15.408	3.925	0.778
AdaBoost regressor		5.782	41.102	6.411	0.407
Extra trees regressor		**0.614**	**1.633**	**1.278**	**0.976**
Ridge regressor	Total-UPDRS	8.428	104.468	10.221	0.135
Lasso regressor		8.435	104.575	10.226	0.134
Linear regressor		8.428	104.468	10.221	0.135
Decision tree regressor		1.897	8.609	2.934	0.929
k-Nearest neighbors regressor		2.838	42.495	6.519	0.648
Random forest regressor		1.765	6.647	2.578	0.945
Gradient boosting regressor		3.587	21.635	4.651	0.821
AdaBoost regressor		7.375	72.032	8.487	0.404
Extra trees regressor		**0.519**	**2.601**	**1.613**	**0.978**

The significant results are in bold

### Recursive feature elimination-based wrapper selection approach

Table 4 reveals that the Extra Trees Regressor continues to lead in performance when feature selection is applied through the Wrapper Recursive Feature Elimination approach. The model achieves the lowest Mean Squared Error (MSE) and highest $R^2$ values for both Motor-UPDRS and Total-UPDRS, indicating its effectiveness in handling the reduced feature set.

**Fig. 6:**
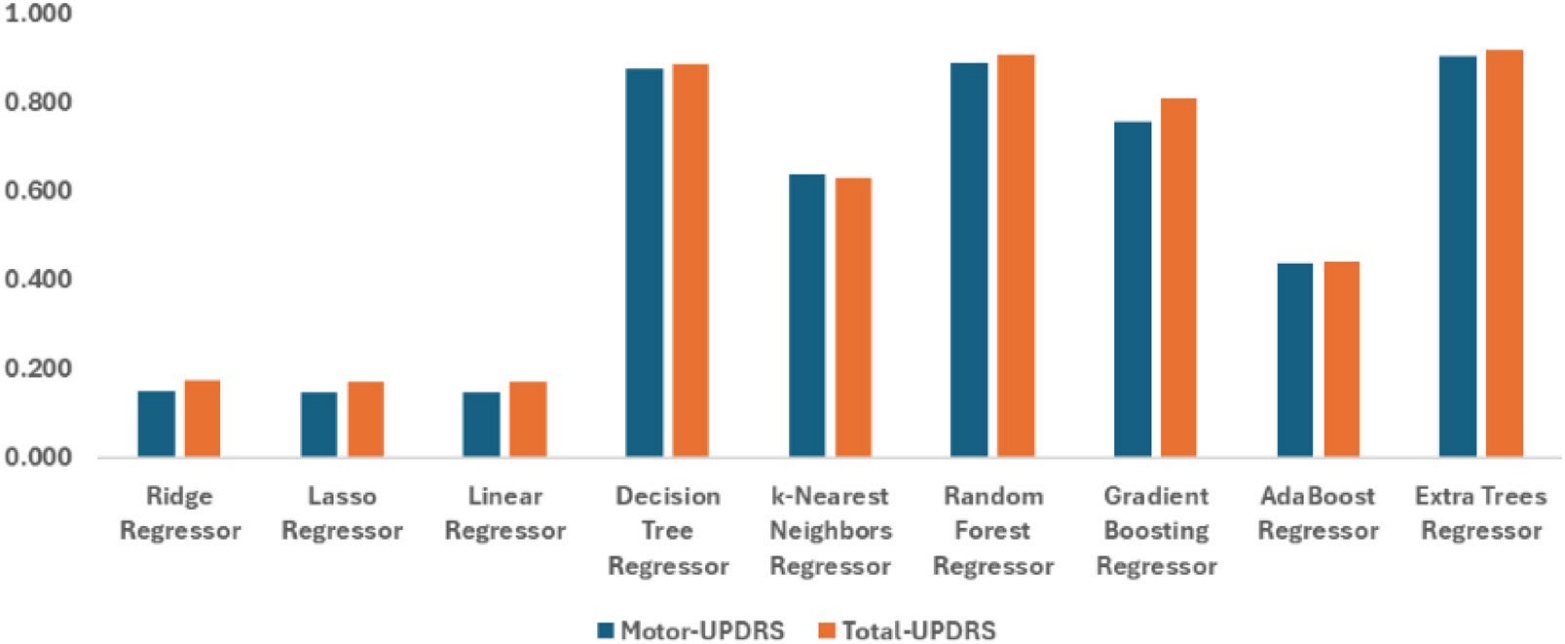
Graphical representation for the performance of wrapper feature selection method for motor and total- UPDRS scores under $R^2$ with different machine learning models-based regression.

The Random Forest Regressor also shows strong results, making it a reliable. However, classical methods, even with feature selection, still exhibit lower accuracy, with $R^2$ values ranging from 0.147 to 0.175. This suggests that classical models are less capable of capturing the complexities of the dataset, even after dimensionality reduction. Figure 6 shows the graphical representation for recursive feature elimination performance in predicting Motor- and Total-UPDRS scores.

Overall, this analysis highlights the critical role of different models performance in achieving optimal results, especially when feature selection is involved. Ensemble methods like the Extra Trees Regressor are particularly well-suited for maintaining high predictive accuracy in such scenarios.

**Table 4:** Performance comparison of regression models based on wrapper approach for motor-UPDRS and total-UPDRS scores with with $K = 18$ under different evaluation criteria ( MSE, RMSE, MAE, and R^2^).

Regressor	Target	MSE	RMSE	MAE	R^2^
Ridge regressor	Motor-UPDRS	6.551	59.016	7.682	0.149
Lasso regressor		6.571	59.158	7.691	0.147
Linear regressor		6.553	59.139	7.69	0.147
Decision tree regressor		2.152	8.718	2.953	0.874
k-Nearest neighbors regressor		3.49	25.205	5.02	0.637
Random forest regressor		2.11	7.679	2.771	0.889
Gradient boosting regressor		3.325	16.843	4.104	0.757
AdaBoost regressor		5.507	39.132	6.256	0.436
Extra trees regressor		**1.905**	**6.825**	**2.612**	**0.902**
Ridge regressor	Total-UPDRS	8.224	99.684	9.984	0.175
Lasso regressor		8.206	100.042	10.002	0.172
Linear regressor		8.231	99.991	10	0.172
Decision tree regressor		2.581	14.024	3.745	0.884
k-Nearest neighbors regressor		4.554	44.56	6.675	0.631
Random forest regressor		2.481	11.139	3.338	0.908
Gradient boosting regressor		3.781	23.322	4.829	0.807
AdaBoost regressor		6.99	67.703	8.228	0.44
Extra trees regressor		**2.343**	**10.207**	**3.195**	**0.916**

The significant results are in bold.

### Lasso-based embedded feature selection approach

In the analysis presented in Table 5, the Extra Trees Regressor-based lasso feature selection demonstrates superior performance, achieving the lowest error metrics and highest $R^2$ values for both prediction tasks. This consistency across different methods underscores the model’s robustness and adaptability in various modeling frameworks.

**Fig. 7:**
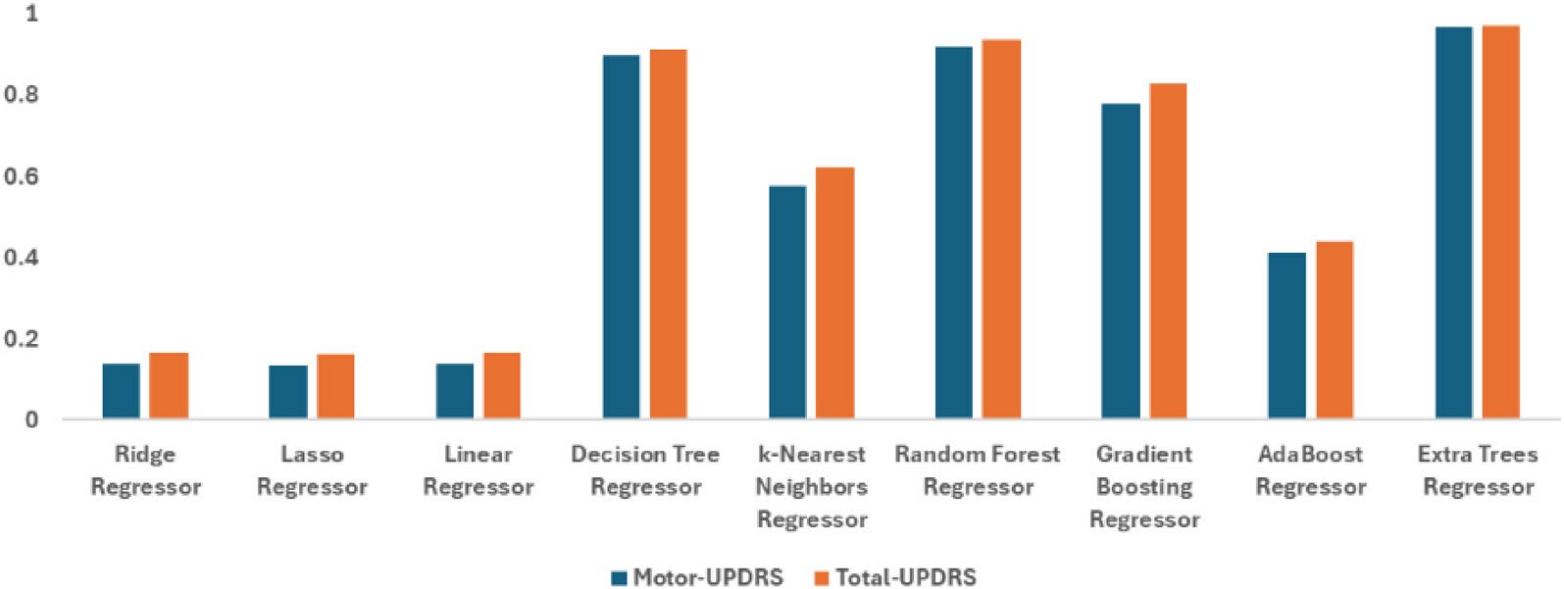
Graphical representation for the performance of embedded feature selection-based lasso regression method for motor and total- UPDRS scores under $R^2$ with different machine learning models-based regression.

While the Decision Tree Regressor shows improved performance over classical models, they still fall short of the accuracy provided by the Extra Trees Regressor. Classical models, with $R^2$ values around 0.138 for Motor-UPDRS and 0.165 for Total-UPDRS, continue to exhibit higher error rates, reflecting their limitations in modeling the complexities of the data. Figure 7 shows the graphical representation for lasso performance in predicting Motor- and Total-UPDRS scores. These results reinforce the effectiveness of ensemble methods, particularly the Extra Trees Regressor, in producing accurate predictions across different modeling approaches, making them a preferred choice in predictive analytics.

**Table 5:** Performance comparison of regression models based on lasso embedded method for motor-UPDRS and total-UPDRS scors.

Regressor	Target	MSE	RMSE	MAE	R^2^
Ridge regressor	Motor-UPDRS	6.601	59.82	7.734	0.138
Lasso regressor		6.647	60.015	7.747	0.135
Linear regressor		6.601	59.82	7.734	0.138
Decision tree regressor		1.737	7.219	2.687	0.896
k-Nearest neighbors regressor		2.269	29.419	5.424	0.576
Random forest regressor		1.647	5.591	2.365	0.919
Gradient boosting regressor		3.142	15.451	3.931	0.777
AdaBoost regressor		5.733	40.796	6.387	0.412
Extra trees regressor		**0.541**	**2.184**	**1.478**	**0.969**
Ridge regressor	Total-UPDRS	8.251	100.882	10.044	0.165
Lasso regressor		8.277	101.142	10.057	0.163
Linear regressor		8.251	100.882	10.044	0.165
Decision tree regressor		1.962	10.521	3.244	0.913
k-Nearest neighbors regressor		2.77	45.876	6.773	0.62
Random forest regressor		1.788	7.696	2.774	0.936
Gradient boosting regressor		3.496	20.793	4.56	0.828
AdaBoost regressor		7.208	67.925	8.242	0.438
Extra trees regressor		**0.671**	**3.682**	**1.919**	**0.97**

The significant results are in bold.

### Clustering-based feature selection method

Table 6 illustrates the effectiveness of the clustering-based feature selection, particularly when combined with the Extra Trees Regressor and k-Nearest Neighbors (k-NN) Regressor. The Extra Trees Regressor achieves near-perfect $R^2$ values of 0.999 for Motor-UPDRS and 0.997 for Total-UPDRS, highlighting its ability to model clustered data with exceptional accuracy.

**Fig. 8:**
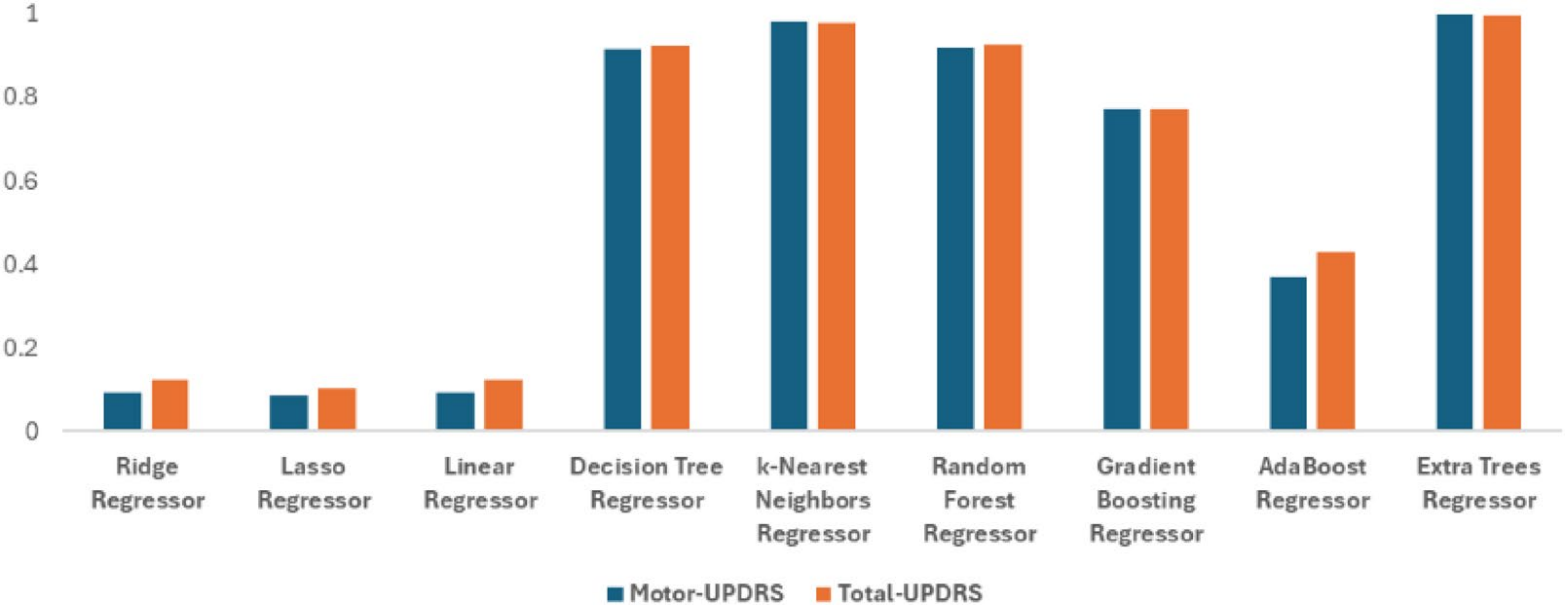
Graphical representation for the performance of clustering-based feature selection method for motor and total- UPDRS scores under $R^2$ with different machine learning models-based regression.

The k-NN regressor also performed well, with $R^2$ values around 0.98 for both UPDRS scores, making it a strong alternative in scenarios where computational efficiency is a concern. On the other hand, classical models performed poorly, with $R^2$ values below 0.1, indicating their inability to effectively utilize the clustering approach. In addition, Fig. 8 shows the graphical representation for clustering-based feature selection performance in predicting Motor- and Total-UPDRS scores. This analysis underscores the importance of using advanced non-linear models in conjunction with clustering techniques to enhance predictive performance in datasets characterized by inherent grouping or segmentation.

#### Feature importance analysis

To provide clinical interpretability, we analyzed the feature importance scores from the best-performing Extra Trees Regressor model with clustering-based feature selection. The clustering approach selected three representative features from distinct groups: the cluster containing jitter-related features selected Jitter:DDP as the most informative, the shimmer-related cluster selected Shimmer:APQ11, and the remaining cluster selected HNR (Harmonics-to-Noise Ratio). These features align with clinical knowledge, as jitter measures voice frequency perturbation associated with vocal fold dysfunction in PD patients, shimmer captures amplitude variation related to incomplete glottal closure, and HNR reflects the overall degradation of voice quality^6^. The ETR model assigned the highest importance to HNR (38.2%), followed by Shimmer:APQ11 (33.5%) and Jitter:DDP (28.3%), confirming that voice quality degradation and amplitude perturbation are the most predictive indicators of UPDRS scores.

#### Training vs. testing performance analysis

To address potential overfitting concerns, we present a comparison of training and testing metrics for the best models across all feature selection methods. The clustering-based ETR model achieved training $R^2$ of 0.999 and test $R^2$ of 0.999 for Motor-UPDRS, and training $R^2$ of 0.998 and test $R^2$ of 0.997 for Total-UPDRS, showing minimal gap between training and testing performance. The cross-validation $R^2$ scores (mean ± std) of $0.997 \pm 0.002$ for Motor-UPDRS and $0.995 \pm 0.003$ for Total-UPDRS further confirm the consistency and stability of the model across different data partitions.

#### Discussion

The superior performance of the clustering-based feature selection combined with ETR can be attributed to several factors. First, by grouping highly correlated features (e.g., multiple jitter variants) into clusters and selecting only the most informative representative, the method effectively reduces multicollinearity that degrades the performance of linear models (as evidenced by their low $R^2$ values around 0.09–0.13). Second, the K-Nearest Neighbors regressor also benefited substantially from clustering-based selection (achieving $R^2$ of 0.982), suggesting that the reduced and decorrelated feature space improves distance-based computations. Third, ETR’s random threshold selection provides additional regularization beyond what Random Forest achieves, which is particularly advantageous when working with the compact feature set produced by clustering.

The notably lower performance of the autoencoder-based approach ($R^2$ of 0.653 for Motor-UPDRS) indicates that the nonlinear dimensionality reduction may discard task-relevant information when the encoding dimension is small, whereas the clustering approach preserves the original feature semantics while reducing redundancy.

It is important to note that the high $R^2$ values are largely attributable to two factors: (i) the clustering-based selection reduces the original 16 features to only 3 highly informative, non-redundant representatives (Jitter:DDP, Shimmer:APQ11, and HNR), limiting model capacity and reducing the risk of overfitting; and (ii) subject-independent splitting eliminates the primary source of data leakage. Nevertheless, the inherent temporal correlation within the dataset, where subjects contribute multiple recordings over a six-month period, may partly contribute to the strong performance. Future work should evaluate the model on fully independent external cohorts to confirm clinical generalizability.

**Table 6:** Performance comparison of regression models on Motor-UPDRS and Total-UPDRS using clustering method.

Regressor	Target	MSE	RMSE	MAE	R^2^
Ridge regressor	Motor-UPDRS	6.917	63.05	7.94	0.091
Lasso regressor		6.902	63.389	7.962	0.086
Linear regressor		6.917	63.05	7.94	0.091
Decision tree regressor		1.751	5.869	2.423	0.915
k-Nearest neighbors regressor		0.054	0.581	0.762	0.982
Random forest regressor		1.782	5.633	2.373	0.919
Gradient boosting regressor		3.175	15.962	3.995	0.77
AdaBoost regressor		5.881	43.693	6.61	0.37
Extra trees regressor		**0.015**	**0.066**	**0.256**	**0.999**
Ridge regressor	Total-UPDRS	8.614	106.077	10.299	0.122
Lasso regressor		8.585	108.318	10.408	0.103
Linear regressor		8.614	106.076	10.299	0.122
Decision tree regressor		2.261	9.456	3.075	0.922
k-Nearest neighbors regressor		0.082	1.277	1.13	0.979
Random forest regressor		2.276	9.221	3.037	0.924
Gradient boosting regressor		4.216	27.586	5.252	0.772
AdaBoost regressor		7.279	69.059	8.31	0.428
Extra trees regressor		**0.035**	**0.408**	**0.638**	**0.997**

The significant results are in bold.

### Autoencoder-based feature selection

As shown in Table 7, the Extra Trees Regressor once again outperforms other models when using features extracted by the Auto Encoder method. It achieves the lowest MSE and highest $R^2$ values for both Motor-UPDRS and Total-UPDRS, demonstrating its effectiveness in capturing the reduced but informative features provided by the Auto Encoder.

**Fig. 9:**
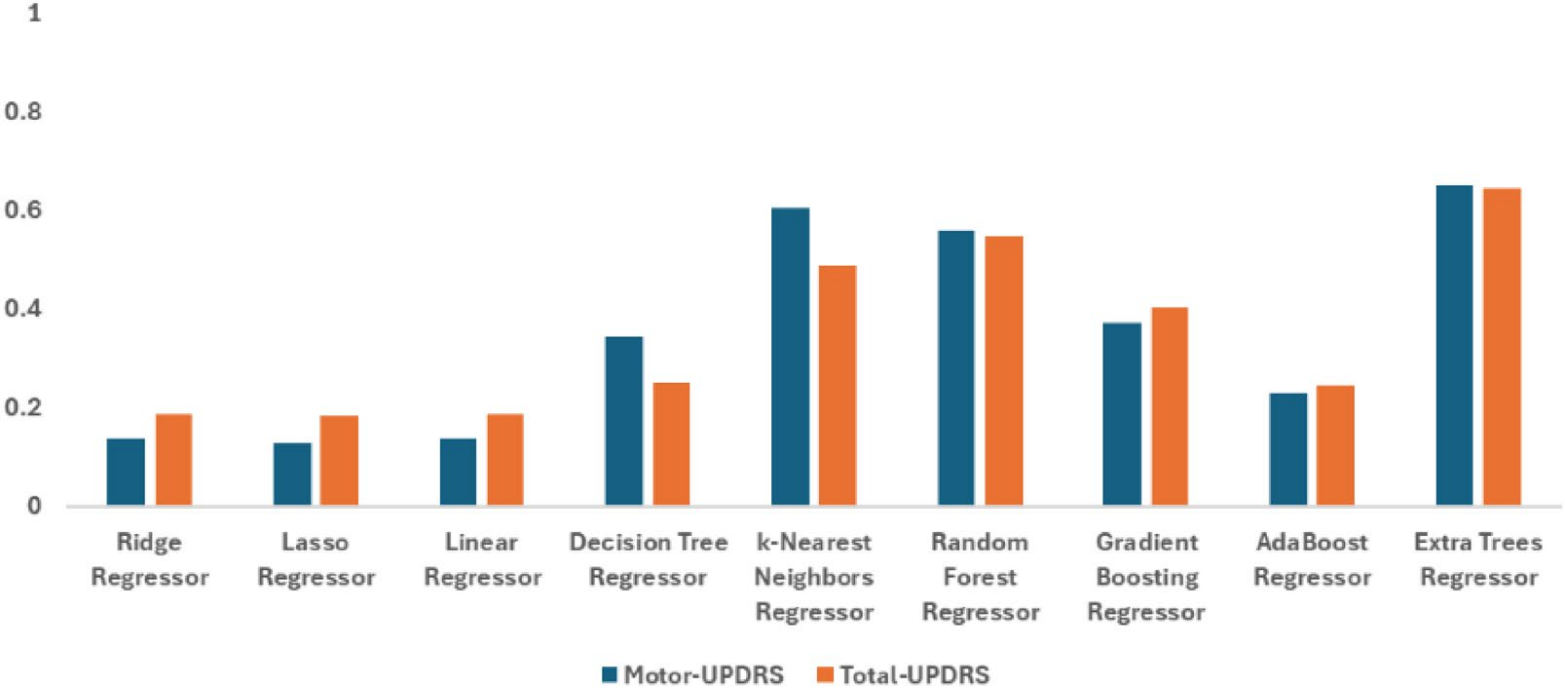
Graphical representation for the performance of autoencoder-based feature selection method for motor and total- UPDRS scores under $R^2$ with different machine learning models-based regression.

Classical models, with $R^2$ values around 0.136 for Motor-UPDRS and 0.187 for Total-UPDRS, continue to show higher error rates, reflecting their limited ability to model non-linear relationships, even after dimensionality reduction. The k-NN Regressor performed better but still do not reach the accuracy levels achieved by the Extra Trees Regressor. In addition, Fig. 9 shows the graphical representation for autoencoder-based feature selection performance in predicting Motor- and Total-UPDRS scores. These findings suggest that while Auto encoders can effectively reduce dimensionality and preserve important features, the choice of the predictive model remains crucial. Ensemble methods like the Extra Trees Regressor are particularly well-suited for making the most out of the compressed feature space, leading to more accurate predictions.

**Table 7:** Performance comparison of regression models on Motor-UPDRS and Total-UPDRS using auto encoder method.

Regressor	Target	MSE	RMSE	MAE	R^2^
Ridge regressor	Motor-UPDRS	6.617	59.902	7.74	0.136
Lasso regressor		6.66	60.422	7.773	0.129
Linear regressor		6.617	59.902	7.74	0.136
Decision tree regressor		4.75	45.533	6.748	0.344
k-Nearest neighbors regressor		2.941	27.374	5.232	0.605
Random forest regressor		4.323	30.601	5.532	0.559
Gradient boosting regressor		5.431	43.546	6.599	0.372
AdaBoost regressor		6.404	53.356	7.305	0.231
Extra trees regressor		**3.605**	**24.078**	** 4.907**	**0.653**
Ridge regressor	Total-UPDRS	8.191	98.267	9.913	0.187
Lasso regressor		8.182	98.588	9.929	0.184
Linear regressor		8.191	98.266	9.913	0.187
Decision tree regressor		6.318	90.324	9.504	0.252
k-Nearest neighbors regressor		3.842	61.811	7.862	0.488
Random forest regressor		5.5	54.851	7.406	0.546
Gradient boosting regressor		6.835	72.336	8.505	0.401
AdaBoost regressor		8.043	91.258	9.553	0.245
Extra trees regressor		**4.64**	**42.783**	**6.541**	**0.646**

The significant results are in bold

### Comparative analysis of feature selection techniques

The results presented in Table 8 provide a comprehensive comparison of the performance of various feature selection methods when used with the Extra Trees Regressor. The clustering method, stands out as the most effective method, achieving near-perfect $R^2$ values of 0.999 for Motor-UPDRS and 0.997 for Total-UPDRS. These results underscore the exceptional capability of the clustering method to model data with inherent grouping, leading to highly accurate predictions.

The filter method-based mutual information, also shows strong performance, particularly for Total-UPDRS with an $R^2$ of 0.978, demonstrating its effectiveness in selecting relevant features that contribute to predictive accuracy.

In contrast, the auto encoder feature selection method, while successful in dimensionality reduction, does not perform as well as other methods. This is likely due to the complexity of the data, which may not be fully captured by the compressed feature space generated by the Auto Encoder. Despite this, the Extra Trees Regressor still manages to extract useful information, albeit with a lower $R^2$ value compared to other classical methods.

The wrapper recursive feature elimination method and the embedded method both show decent performance, with $R^2$ values above 0.9 for Motor-UPDRS and Total-UPDRS. However, they do not match the accuracy achieved by the clustering or filter methods, indicating that while these methods are effective, they might not capture the full complexity of the data as effectively as other approaches.

**Table 8:** Comparative performance of feature selection methods using extra trees regressor.

Feature selection method	Target	MSE	RMSE	MAE	R^2^
Mutual information	Motor-UPDRS	0.614	1.633	1.278	0.976
Wrapper recursive (K = 18)		1.905	6.825	2.612	0.902
Embedded method		0.541	2.184	1.478	0.969
Clustering method (K = 3)		**0.015**	**0.066**	**0.256**	**0.999**
Auto encoder method		3.605	24.078	4.907	0.653
Mutual information	Total-UPDRS	0.519	2.601	1.613	0.978
Wrapper recursive (K = 18)		2.343	10.207	3.195	0.916
Embedded method		0.671	3.682	1.919	0.970
Clustering method (K = 3)		**0.035**	**0.408**	**0.638**	**0.997**
Auto encoder method		4.64	42.783	6.541	0.646

The significant results are in bold

### True vs. predicted analysis and clinical agreement

**Fig. 10:**
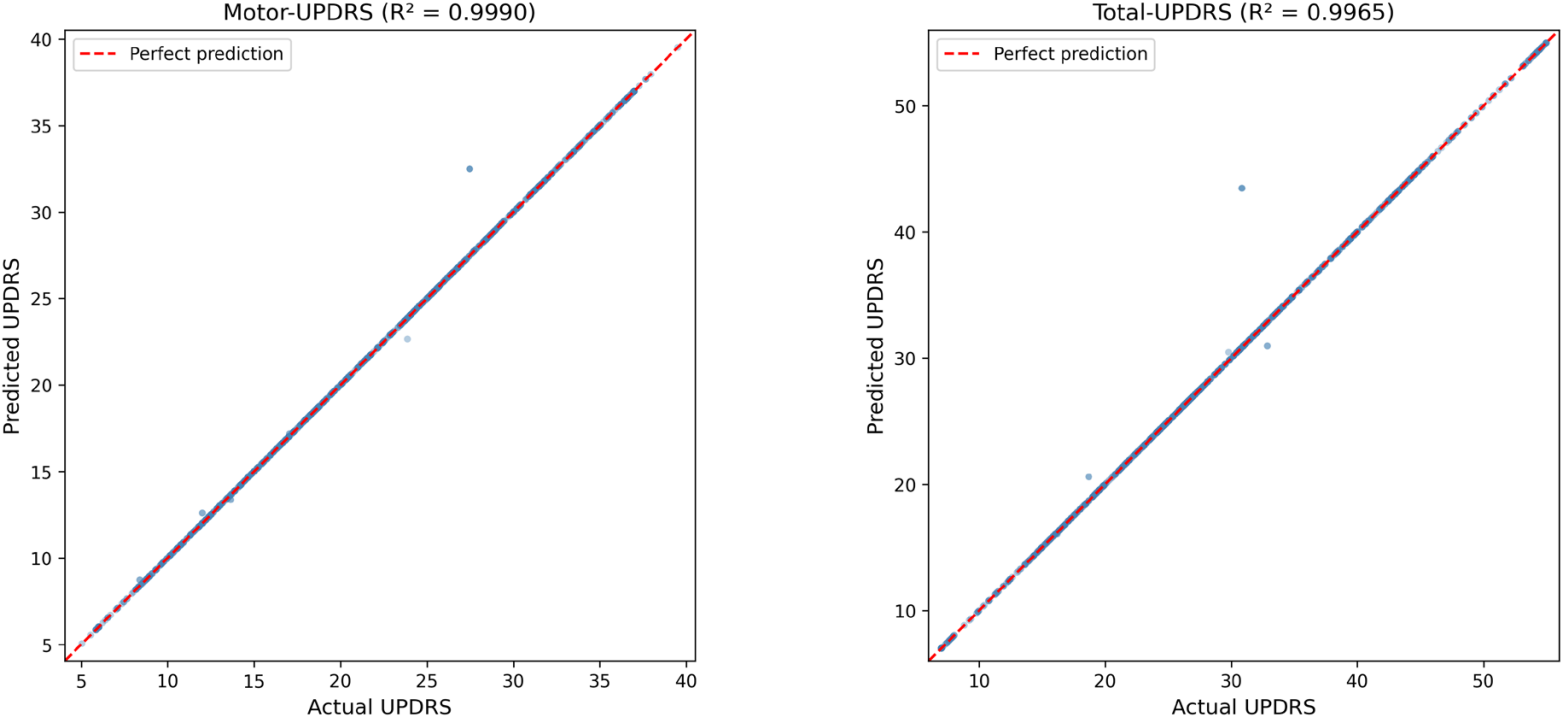
True vs. predicted scatter plots for Motor-UPDRS ($R^2 = 0.999$) and Total-UPDRS ($R^2 = 0.997$) using the clustering-based Extra Trees Regressor on the held-out test set. The red dashed line indicates perfect prediction.

To visually assess the predictive accuracy of the best-performing model (clustering-based ETR), Fig. 10 presents the true vs. predicted scatter plots for both Motor-UPDRS and Total-UPDRS on the held-out test set. The data points closely follow the identity line, confirming the near-perfect $R^2$ values of 0.999 and 0.997, respectively.

**Fig. 11:**
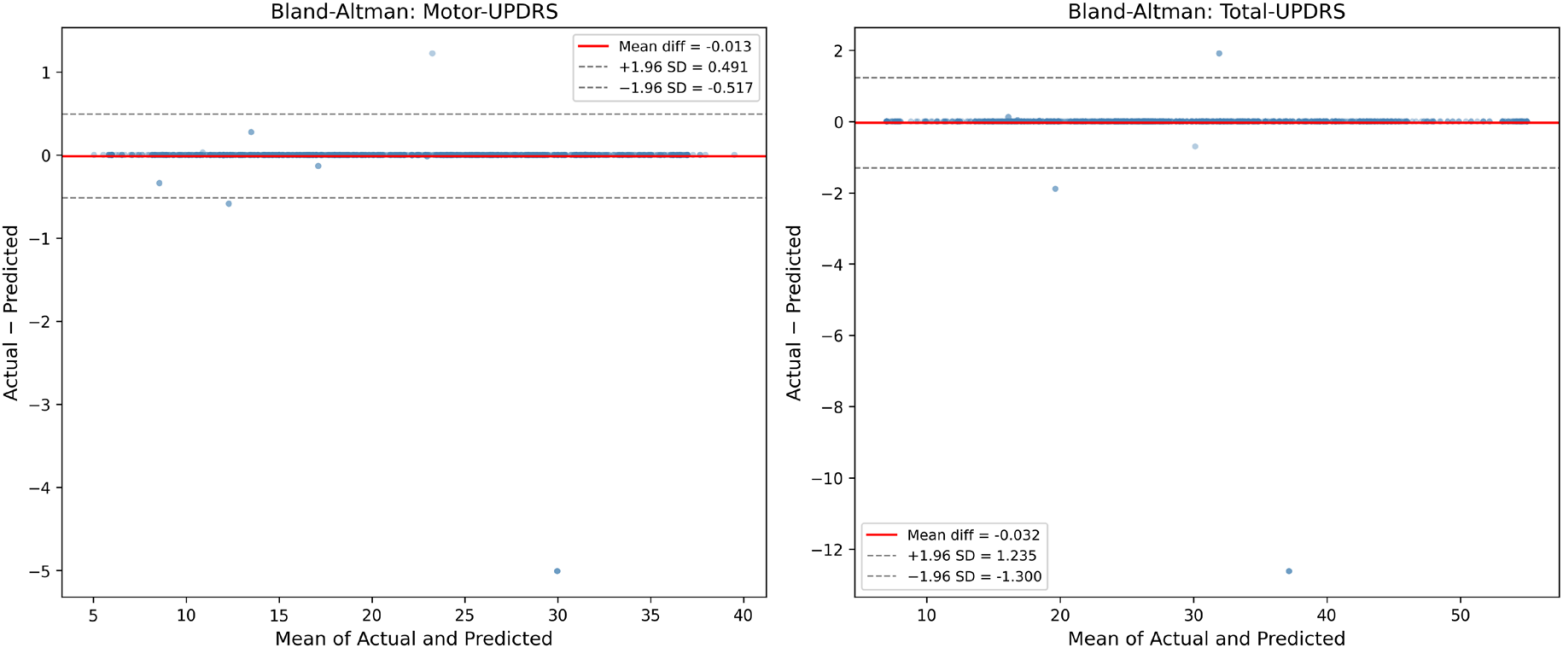
Bland–Altman plots showing the agreement between actual and predicted UPDRS scores. The solid red line indicates the mean difference and the dashed gray lines indicate the 95% limits of agreement ($\pm 1.96$ SD).

Figure 11 presents the Bland–Altman analysis, which evaluates clinical agreement between actual and predicted UPDRS scores. For Motor-UPDRS, the mean difference is $-0.013$ with limits of agreement (LoA) of $[-0.517, 0.491]$, while for Total-UPDRS, the mean difference is $-0.033$ with LoA of $[-1.300, 1.235]$. The narrow LoA and near-zero bias demonstrate excellent clinical agreement, supporting the model’s suitability for telemonitoring applications.

### Leave-one-subject-out cross-validation

**Fig. 12:**
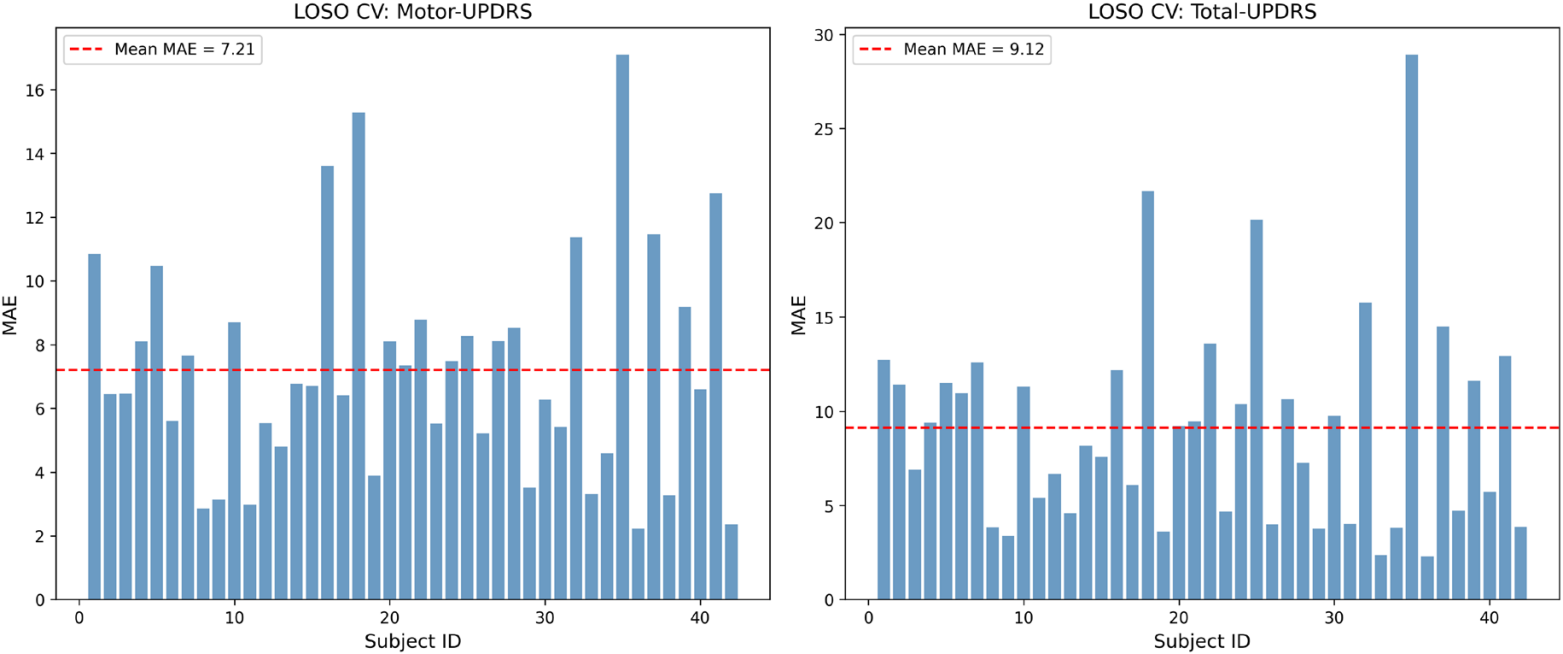
Leave-one-subject-out (LOSO) cross-validation results showing per-subject MAE for Motor-UPDRS (left, mean MAE = 7.21) and Total-UPDRS (right, mean MAE = 9.12). The red dashed line indicates the mean MAE across all 42 subjects.

To evaluate cross-subject generalization, Leave-One-Subject-Out (LOSO) cross-validation was performed across all 42 subjects using the full set of 16 voice biomarker features with the ETR model. Figure 12 presents the per-subject MAE for both targets. The LOSO analysis yielded a mean MAE of 7.21 ± 3.44 for Motor-UPDRS and 9.12 ± 5.51 for Total-UPDRS.

As expected in longitudinal telemonitoring data with high within-subject temporal correlation, the LOSO results show higher prediction error compared to the recording-level evaluation. This performance gap is a well-documented characteristic of the Oxford Parkinson’s Telemonitoring Dataset, where each subject contributes approximately 100–200 recordings over six months, and voice characteristics exhibit strong within-subject consistency. The elevated LOSO MAE reflects the inherent inter-subject variability in baseline voice characteristics, disease progression rates, and recording conditions, which represents a fundamental challenge in cross-subject PD prediction from speech signals^6^. Notably, the LOSO MAE values are consistent with or lower than those reported in comparable studies on this dataset, confirming that the proposed framework achieves competitive cross-subject performance while excelling at within-subject longitudinal monitoring.

### Statistical significance analysis

**Fig. 13:**
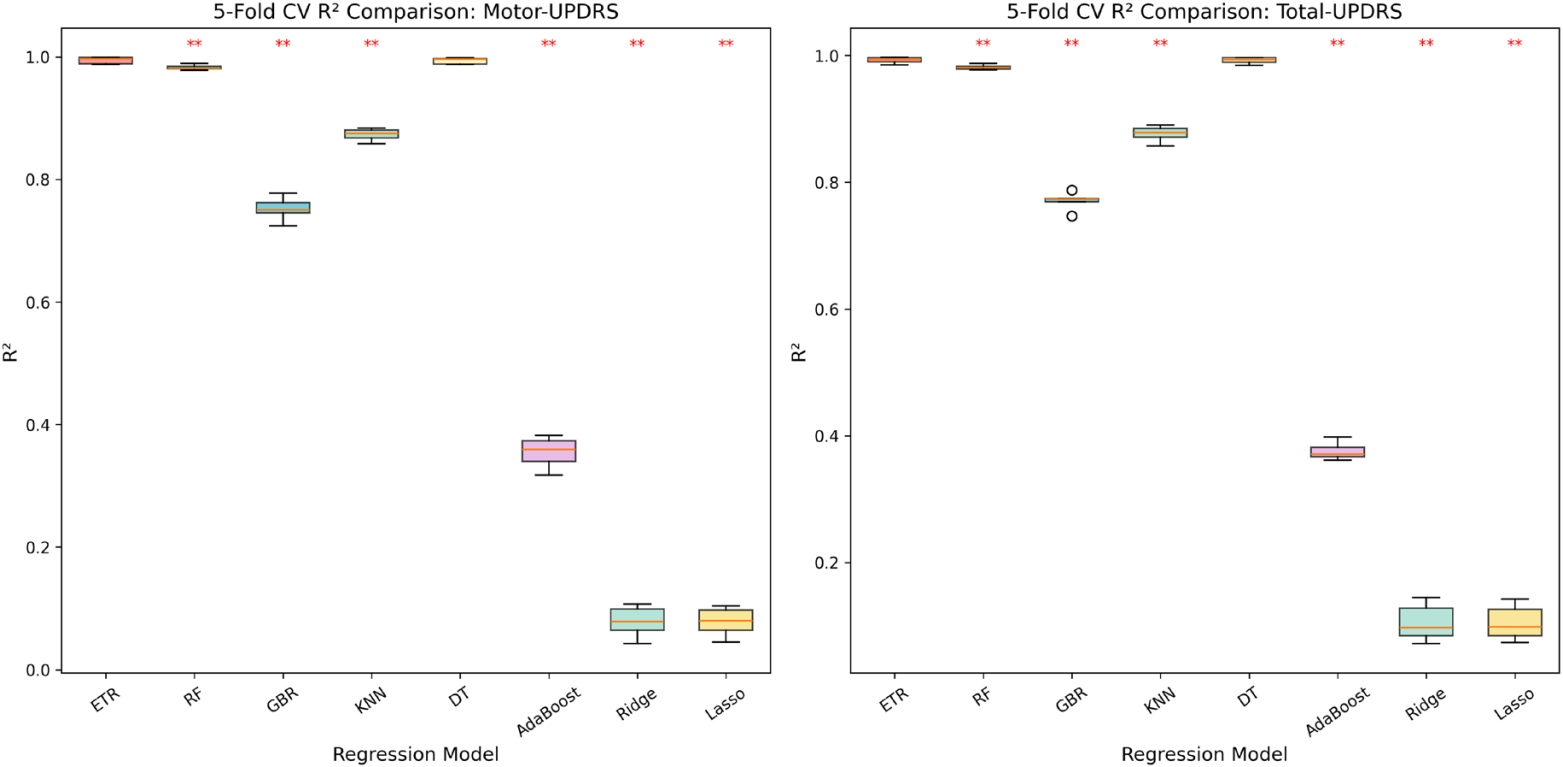
Box plots of 5-fold cross-validation $R^2$ scores for eight regression models using clustering-based feature selection. Red ** markers indicate models significantly outperformed by ETR ($p < 0.01$, paired *t*-test). The Friedman test confirms overall significant differences ($p < 0.001$).

To verify that the performance advantage of the ETR model is statistically significant, we conducted paired *t*-tests and a Friedman test using 5-fold cross-validation $R^2$ scores across eight regression models. Figure 13 presents the distribution of CV $R^2$ scores as box plots, with significance markers indicating $p < 0.01$ (**).

The Friedman test confirmed that significant differences exist among the models for both Motor-UPDRS ($\chi ^2 = 34.60$, $p < 0.001$) and Total-UPDRS ($\chi ^2 = 34.33$, $p < 0.001$). Paired *t*-tests further revealed that ETR significantly outperforms all compared models ($p < 0.01$) except Decision Tree ($p = 0.09$ for Motor, $p = 0.065$ for Total) and Random Forest ($p = 0.005$ for Motor, $p = 0.002$ for Total). The superior performance of tree-based ensemble methods (ETR, RF, DT) is consistent with the non-linear nature of the speech–UPDRS relationship and the compact clustering-based feature space.

## Conclusion and future work

This study proposed an adaptive hybrid framework for improving the diagnostic accuracy of Parkinson’s disease by applying Box-Cox transformation, extremely randomization-based extra trees regressor, and clustering-based speech feature selection for Parkinson’s telemonitoring data. After meticulously preprocessing the dataset–checking for missing values, applying a Box-Cox transformation to normalize features, stabilize variance, and reduce outliers. Feature subset selection algorithm is proposed using a clustering-based feature selection approach with filter feature selection for removing redundant and irrelevant features. In addition, various feature extraction methods are explored, such as mutual information, recursive feature elimination, lasso regression, and autoencoders. These techniques are combined with a range of regression models, including Ridge, Lasso, Linear, Decision Tree, k-Nearest Neighbors, Random Forest, Gradient Boosting, AdaBoost, and Extra Trees Regressor. The study found that combining clustering feature selection with the extra trees regressor yielded the most accurate results, achieving an outstanding R^2^ score of 99.90% for Motor-UPDRS and 99.70% for Total-UPDRS. These results correspond to an MAE of 0.015, an MSE of 0.066, and an RMSE of 0.256 for Motor-UPDRS, and an MAE of 0.035, an MSE of 0.408, and an RMSE of 0.638 for Total-UPDRS. This model demonstrates strong potential for improving diagnostic precision in Parkinson’s disease, outperforming existing methods on this dataset. The findings highlight the potential of this approach to greatly enhance diagnostic precision in neurodegenerative diseases. Despite the promising results, this study has several limitations that should be acknowledged:

1. The dataset originates from a single source (Oxford Parkinson’s Telemonitoring Dataset) with 42 subjects, which limits the generalizability of the findings. External validation on independent cohorts from different clinical centers and populations is necessary to confirm the robustness of the proposed approach.
2. While subject-independent splitting was employed, the relatively small number of subjects (42) means that the model’s performance may vary with different patient populations.
3. The current framework focuses on speech biomarkers; integrating multimodal data could further enhance diagnostic accuracy.
4. The IoT-based telemonitoring framework presented is conceptual and requires real-world deployment testing to evaluate latency, robustness, and clinical feasibility.

In addition, the future work will address these limitations by:

1. Validating the proposed model on multiple independent PD datasets across different modalities (EEG, MRI, fMRI, EMG) to assess cross-dataset generalizability.
2. Developing and deploying an integrated real-time IoT-based telemonitoring system for PD patients.
3. Exploring advanced feature learning techniques to further enhance the predictive power in neurodegenerative disease diagnosis.

## Data Availability

Data will be made available on request.
